# Use of pIVEX plasmids for protein overproduction in *Escherichia coli*

**DOI:** 10.1186/1475-2859-4-18

**Published:** 2005-06-02

**Authors:** Julie Rogé, Jean-Michel Betton

**Affiliations:** 1 Unité de Repliement et Modélisation des Protéines Institut Pasteur CNRS-URA2185 28, rue du Docteur Roux 75724 Paris Cedex 15, France

## Abstract

**Background:**

The pIVEX plasmids are vectors optimized for expression in the Rapid Translation System (RTS) cell-free system under control of bacteriophage T7 transcription elements. Even if these plasmids are intended for use *in vitro*, it is usually worthwhile to compare both cell-free and bacterial expression from the same genetic construct. However, some RTS users encountered problems when they introcuded these plasmids into *Escherichia coli *host strains producing the T7 RNA polymerase.

**Results:**

We verified that difficulties in transforming the commonly used BL21(λDE3) strain with pIVEX arose from the presence of a strong T7 promoter combined with a high-copy number plasmid, independent of gene expression. When these vectors were introduced into this strain harboring a compatible plasmid carrying the lactose repressor (*lacI*), we improved the transformation efficiency by 4 orders of magnitude. Moreover, we designed a transformation protocol that allows, after induction, the overproduction of pIVEX-encoded proteins in the BL21(λDE3) strain.

**Conclusion:**

Using the correct plasmid/host combination and transformation-expression protocol, we could directly compare overproduction of the same pIVEX-encoded proteins from both *in vivo *and *in vitro *expression systems.

## Background

Recent developments in cell-free systems offer new and promising possibilities for producing recombinant proteins [1]. The RTS, commercialized by Roche, is an exchange cell-free system with improved productivity [2]. The continuous supply of consumable substrates and removal of reaction products provide a yield of several milligrams of protein. This system uses bacteriophage T7 RNA polymerase to perform transcription, while an enriched *E. coli *S30 extract provides the translational machinery. Thus, protein production in RTS requires a preliminary cloning step of the target gene into a vector, downstream of the T7 promoter. For this purpose, the pIVEX family of expression plasmids has been optimized for *in vitro *use. They include a T7 promoter comprising the T7 gene 10 translation enhancer [3], an efficient prokaryotic Shine-Dalgarno sequence with an optimum distance to the start codon, a multiple cloning site, and a T7 terminator which prevents 3'-terminal exonucleolytic degradation of the mRNA. These vectors are very convenient since multiple cloning sites were designed to allow gene fusions with several tags either at the N- or C-terminal of target proteins by conserving the same restriction strategy and reading frame compatibility.

Some investigations revealed that less than optimal expression in this cell-free system could be explained by the presence of stable secondary structures in mRNAs [4]. Indeed, transcription levels are kept very high due to the highly active T7 RNA polymerase. Consequently, no real coupling of transcription and translation can take place *in vitro*, and unprotected mRNAs may form stable secondary structures, notably in the translation initiation region that inhibit ribosome binding and limit expression levels. There are mRNA folding algorithms that can predict such unfavourable intramolecular secondary structures, but they do not give information about expression levels. Although similar regulatory mechanisms exist in *E. coli *[5], it is simple and useful to assess *in vivo *expression levels from a pIVEX plasmid which gave poor protein yields *in vitro*. The influence of mRNA secondary structures on translation is not identical in both contexts. However, transformation of the most widely used T7 RNA polymerase-producing BL21(λDE3) strain [6] by pIVEX is impaired because the absence of the *lacI *gene, coding the lactose repressor, in these high copy number plasmids. Here, we report how to solve this problem by designing a simple protocol with a compatible plasmid carrying the lactose repressor gene. This method allows the direct comparison of *in vitro *and bacterial expression from pIVEX vectors.

## Results and Discussion

Unlike other T7 promoter-based vectors, the pIVEX do not contain a *lac *operator sequence downstream of the T7 promoter. Since expression from pIVEX is not repressed by LacI, they do not contain the corresponding gene. In fact, first attempts to transform, by a standard chemical procedure, the BL21(λDE3) strain with different pIVEX failed since no transformant was obtained on LB ampicillin-agar plates. On the assumption that basal expression from pIVEX may have adverse effects on bacterial growth, we decided to test various plasmid/host combinations in order to control more tightly transcription both at the *lacUV5 *promoter of the T7 RNA polymerase gene in the host chromosome, and consequently at the T7 promoter in the pIVEX plasmid. Furthermore, other investigations indicated that the transformation efficiency of this *E. coli *B strain could be critical [7]. Therefore, we electroporated freshly BL21(λDE3) competent cells containing either the pLysS plasmid encoding T7 lysozyme [8], a natural inhibitor of T7 RNA polymerase, or the pDIA17 plasmid [9] harboring the *lacI *gene. Both resident plasmids are chloramphenicol resistant and compatible with pIVEX since they carry the origin of replication from plasmid p15A. To facilitate the analysis of expression among the recombinants, a pIVEX-GFP plasmid encoding the green fluorescence protein, GFP, was used for fluorescence screening of single colonies on agar plates. As a control for toxic expression, the same strains were also transformed with an empty pIVEX plasmid (pIVEX2.4d).

The results presented in Table 1 show that 1) the presence of the cloned gene coding GFP into pIVEX has no influence on transformation efficiency, 2) pLysS did not increase the tolerance of BL21(λDE3) for either of the pIVEX plasmids employed, and 3) in contrast, pDIA17 increases the transformation efficiency by 4 orders of magnitude. These results suggest that the basal production of T7 RNA polymerase in BL21(λDE3) was incompatible with a high-copy number plasmid which does not encode the *lacI *gene, even without a cloned gene downstream the T7 promoter. Yet, a substantial increase in lactose repressor from pDIA17 in BL21(λDE3) cells is sufficient to permit the presence of pIVEX.

**Table 1:** Transformation efficiencies of pIVEX vectors into BL21(DE3) strain

Resident plasmid (p15A derivative)	Incoming plasmid (pIVEX vector)	Transformation efficiency (transformants per μg of DNA)^a^	Colony fluorescence^b^
none	pIVEX2.4d	3.1 × 10^4 ^± 0.8	-
none	pIVEX-GFP	3.5 × 10^4 ^± 0.6	85 % F 15 % NF
pLysS	pIVEX2.4d	4.5 × 10^4 ^± 0.8	-
pLysS	pIVEX-GFP	4.6 × 10^4 ^± 0.7	90 % F 10 % NF
pDIA17	pIVEX2.4d	7.5 × 10^8 ^± 1.5	-
pDIA17	pIVEX-GFP	8.2 × 10^8 ^± 2.5	100 % F

a Results are mean of three experiments ± SD on LB plates containing antibiotics.

b Transformation plates containing 100 to 200 colonies were observed under UV illumination, NF; non-fluorescent, F; fluorescent

Furthermore, the production of GFP could be directly assessed from the transformation plates. At 37°C in the absence of IPTG, GFP production from pIVEX-GFP, after overnight cell growth on solid LB medium, was sufficient to give fluorescent colonies under these conditions. These high levels of leaky expression is puzzling, but it has been shown that when uninduced BL21(λDE3) cells are grown to stationary phase in LB medium lacking glucose, the *lacUV5 *promoter is derepressed [10]. Apparently, LacI levels from pDIA17 are sufficient to increase pIVEX tolerance in the competent state, but not to ensure stringent repression in the uninduced stationary state. Examination of transformation plates revealed that 100 % of colonies from BL21(λDE3)/pDIA17 cells were fluorescent, but only 90 % from those bearing pLysS (Table 1). This result suggesting that some transformants lacked GFP production, is in good agreement with a recent report in which the highest production level of a recombinant hemoglobin, from a comparison of both strains, was found in the BL21(λDE3)/pDIA17 strain [11]. Although all transformants obtained with pIVEX-GFP in this strain displayed bright fluorescence on transformation plates, a similar lack of GFP production was observed by subculturing repeatedly these cells in liquid LB medium, containing both ampicillin and chloramphenicol. Indeed, many studies have already reported that poor protein synthesis or complete lack of protein production is a common and frequent problem when using the T7 promoter expression system. Production of toxic proteins causing plasmid instability is the conventional explanation for this observation [8]. However, a recent investigation has suggested that the decrease in protein production levels in the BL21(λDE3) strain is more attributable to chromosomal mutations reducing the level of functional T7 RNA polymerase than to significant plasmid loss or to mutations arising on the plasmid [12]. Consistent with this study, isolation of pIVEX-GFP DNA from the subcultured non-expressing cells and transformation into new competent BL21(λDE3)/pDIA17 cells restored GFP production.

**Figure 1:**
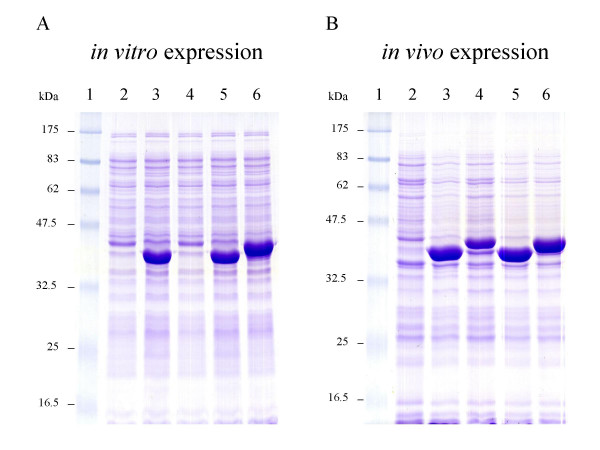
Comparison of MalE overproduction between *in vitro *and *in vivo *expression systems. RTS500 *E. coli *HY extracts (A) and BL21(DE3)/pDIA17 whole cell lysates were separated on 12% SDS-polyacrylamide gel electrophoresis followed by staining with Coomassie blue. Lanes 1, molecular weight marker; lanes 2, pIVEX2.4d; lanes 3, pIV2.1ME; lanes 4, pIV2.2ME; lanes 5, pIV2.3ME; lanes 6, pIV2.4ME.

To compare both *in vitro *and *in vivo *expression, we used the maltose-binding protein (MalE), the soluble receptor for the high-affinity transport of maltose of *E. coli*, as a model protein [13]. Previously, we showed that some additional sequences at the N-terminus of this protein could influence its production level in the RTS cell-free system [14]. The four used pIVEX plasmids allow protein fusion with two different peptide-tags, His- and Strep-tag, either at the N- or C-terminus of MalE [15]. Among these, one construct (pIV2.2ME) yielded only very low levels of protein in the cell-free expression system (Figure 1). Indeed, when the Strep-tag was fused to the N-terminus of MalE, the corresponding protein was undetectable on SDS-polyacrylamide gels stained with Coomassie blue. Since His-tagged MalE at the N-terminus was correctly produced, we hypothesized that the Strep-tag sequence rather than the N-terminal position had a negative influence, probably by forming an unfavorable secondary structure in the translation initiation region of mRNA, which hampers protein synthesis. Because this phenomenon could be minimized inside *E. coli *cells, we examined *malE *expression in BL21(λDE3) from the same four pIVEX plasmids.

To ensure correct cellular protein production, we freshly transformed BL21(λDE3) carrying pDIA17 with the various plasmids, and instead of selecting transformants on LB agar plates supplemented with ampicillin and chloramphenicol, the selection was carried out in liquid LB medium containing both antibiotics. Next day, the saturated cultures were diluted (1:100) into fresh liquid LB medium, supplemented only with ampicillin, and incubated at 30°C. We found that processing the whole population of transformants was less tedious than screening individual colonies for correct expression, and by lowering the growth temperature to 30°C, the copy number of the pUC plasmid was decreased [16]. Subsequently, cultures were induced during the log phase, and cells were harvested 1.5 h after adding IPTG. Following lysis, the steady-state levels of MalE were analyzed by SDS-polyacrylamide gel electrophoresis. As shown in Figure 1, all pIVEX yielded very large amounts of MalE under these conditions. Although expression from pIV2.2ME was lower than from other plasmids, the corresponding protein was easily detectable on the gel stained by Coomassie blue. Thus, in contrast to RTS expression, the N-terminus Strep-tagged MalE is correctly produced in *E. coli *cells. In practice, a successfull cellular expression may help to validate a pIVEX plasmid in which the target gene is correctly introduced between the regulatory elements, but gave no detectable expression *in vitro*.

## Conclusion

Using a correct plasmid/host combination and a simple transformation-expression protocol, we could directly compare the production of the same pIVEX-encoded proteins in two related expression systems, but performed *in vivo *and *in vitro*. With the development of cell-free systems in structural genomic and functional proteomic programs, it will be interesting to systematically investigate, on a large set of proteins, whether expression behaves similarly in both environments.

## Materials and methods

### Bacterial strain and transformation

*E. coli *B strain BL21(λDE3) was obtained from Novagen. Electroporation was performed using an Eppendorf Electroporator 2510. Bacteria were grown in 100 ml LB to A_600 _of 0.7, chilled on ice, and harvested by centrifugation (10 min, 1000 g at 4°C). The pellet was washed twice with 100 ml ice-cold distilled water, and once with 5 ml ice-cold 10 % glycerol. The last bacterial pellet was suspended in a final volume of 500 μl in 10 % glycerol. Aliquots (50 μl) were mixed with 10 ng plasmid DNA in chilled cuvettes (0.2 cm electrode gap). A simple pulse of 12.5 kV/cm was applied and 1 ml of SOC was immediately added. Then, electroporated bacteria were transferred to polypropylene culture tubes and shaken for 1 h at 37°C before either plating on LB plates containing ampicillin (100 μg/ml) and chloramphenicol (25 μg/ml), or diluting into liquid LB medium supplemented with both antibiotics.

### Plasmids

Plasmid pLysS was obtained from Novagen, and pIVEX-GFP, encoding the GFP cycle 3 variant [17], was a generous gift from Cordula Nemetz. Plasmids pIV2.1ME, pIV2.2ME, pIV2.3ME, and pIV2.4ME, carrying the wild-type *malE *gene without signal sequence, under the control of T7 promoter, were previously constructed [14] by subcloning the same PCR product into the corresponding pIVEX2.1MCS, pIVEX2.2bNde, pIVEX2.3MCS, and pIVEX2.4bNde vectors (Roche). Plasmid pDIA17 is a pACYC184 derivative carrying the *lacI *gene under the control of the tetracycline promoter [9].

### Protein synthesis reaction

*In vitro *MalE synthesis was performed in RTS500 *E. coli *HY with the RTS ProteoMaster instrument, essentially as described in the Instruction Manual from Roche. The coupled transcription/translation reactions, initiated by adding 15 μg of pIVEX plasmid DNA, were carried out in 1 ml total volume for 20 hours at 30°C. *In vivo *expression was performed in BL21(λDE3) cells bearing pDIA17 plasmid at 30°C in LB medium supplemented with ampicillin (100 μg/ml). Cultures were induced at an A_600 _of 0.8 by addition of IPTG to a final concentration of 500 μM, and growth was allowed for 1.5 hours after addition of IPTG. Proteins from both *in vitro *and *in vivo *extracts were separated by 12% SDS-polyacrylamide gel and stained by Coomassie blue.

## Abbreviations used

GFP – green fluorescent protein

IPTG – isopropyl – β-D-galactoside

LB – Luria Bertani

MalE – maltose binding protein

PCR – polymerase chain reaction

RTS – Rapid Translation System

SDS – sodium dodecyl sulfate

## Authors' contributions

JR performed bacterial transformations. JMB performed protein synthesis and prepared the manuscript. Both authors read and approved the final manuscript.
